# In Vitro Activity of Cefiderocol and Aztreonam/Avibactam Against Gram-Negative Non-Fermenting Bacteria: A New Strategy Against Highly Antibiotic-Resistant Infectious Agents

**DOI:** 10.3390/antibiotics14080762

**Published:** 2025-07-29

**Authors:** Jan Závora, Václava Adámková, Alžběta Studená, Gabriela Kroneislová

**Affiliations:** 1 Clinical Microbiology and ATB Centre, General University Hospital, 128 08 Prague, Czech Republic; 2 Department of Medical Microbiology, Palacky University, 779 00 Olomouc, Czech Republic

**Keywords:** cefiderocol, aztreonam/avibactam, susceptibility, non-fermenting gram-negative bacilli, antimicrobial resistance, immunocompromised patients

## Abstract

Background/Objectives: Non-fermenting Gram-negative bacilli (NFGNB) represent a significant clinical challenge due to their intrinsic and acquired resistance, particularly in immunocompromised patients. Infections cause by NFGNB are associated with high morbidity and mortality, especially among patients with cystic fibrosis and hematologic malignancies. This study aimed to assess the in vitro susceptibility of clinically relevant NFGNB isolates to two newer antibiotics, cefiderocol and aztreonam/avibactam, and an established antibiotic, trimethoprim/sulfamethoxazole. Methods: This retrospective, monocentric study analysed 94 NFGNB isolates (30 *Pseudomonas aeruginosa*, 30 *Acinetobacter* sp., 24 *Stenotrophomonas maltophilia*, and 10 *Burkholderia cepacia* complex). Susceptibility testing for cefiderocol, aztreonam/avibactam, and trimethoprim/sulfamethoxazole was conducted using gradient strip method. MIC values were interpreted using EUCAST breakpoints, ECOFFs, or alternative criteria when necessary. Results: All *S. maltophilia* isolates were susceptible to cefiderocol (FCR) and aztreonam/avibactam (A/A) based on ECOFFs, with one strain resistant to trimethoprim–sulfamethoxazole (COT). *Burkholderia cepacia* complex strains also showed high susceptibility to FCR, with only one isolate exceeding the ECOFF for A/A, and 20% resistant to COT. All *Acinetobacter* sp. isolates were susceptible to FCR; however, most MIC values clustered at or just below the ECOFF value. In *P. aeruginosa*, one isolate was resistant to FCR, and three isolates (10%) were resistant to A/A. Interestingly, confirmed carbapenemase producers remained susceptible to both FCR and A/A. Most A/A MIC values for *P. aeruginosa* were just below the ECOFF. Conclusions: Cefiderocol and aztreonam/avibactam demonstrated promising in vitro activity against clinically relevant NFGNB, including carbapenem-resistant strains. These findings support their potential role as therapeutic options for difficult-to-treat infections, particularly in immunocompromised patients.

## 1. Introduction

Since the discovery of antibiotics, the threat of antimicrobial resistance (AMR) is increasing. And now, the reality of the 21st century is that more than 1.3 million deaths annually are directly attributable to antimicrobial resistance. Furthermore, it was famously predicted that this number will globally reach 10 million by 2050 [1].

Recently, antibiotic resistance has been intensively monitored in Enterobacterales; however, non-fermenting Gram-negative bacilli (NFGNB) can also acquire considerable multidrug resistance, representing a remarkable clinical challenge [2].

NFGNB form a group of particularly troublesome bacteria. Infections caused by these pathogens are predominantly healthcare-associated and are associated with higher rates of mortality and morbidity, extended hospitalization, and increased healthcare costs [3]. Members of NFGNB are commonly present in the environment and are associated with moisture, which, in a hospital setting, may include sources such as air conditioning systems or humidifiers. They also tend to colonize artificial materials like catheters, endoscopes, or ventilator components. Apart from acquired resistance, NFGNB also utilize wide variety of intrinsic resistance mechanisms (production of modifying enzymes, efflux pumps, decreased cell wall permeability), which complicate the selection of effective antimicrobial therapy [4].

Even though NFGNB show low to moderate pathogenicity, there are specific patient groups with high risk of developing severe infection with high mortality. Cystic fibrosis patients are the most affected due to the pathogenesis of the disease, selective antibiotic pressure, and the pathogens’ ability to form biofilms [5].

Patients with haematological malignancies also form a substantial group of sensitive individuals. The reasons for higher risk of severe NFGNB infections include frequent contact with healthcare settings, immunosuppressive therapy, selective pressure of prophylactic antibiotics, neutropenia, and therapy-induced mucositis leading to the translocation of resident microbiota into the bloodstream [6,7].

Given the pathogenic potential of NFGNB in immunocompromised patients, along with their extensive intrinsic and increasing acquired antimicrobial resistance, it is necessary to explore new therapeutic options. Cefiderocol and the combination of aztreonam with avibactam are relatively new antibiotics with possible effects against NFGNB. Furthermore, the guidelines of the Infectious Diseases Society of America (IDSA) from 2024 already recommend cefiderocol as one of the options for combination therapy of *Stenotrophomonas maltophilia* infections. Ceftazidime/avibactam combined with aztreonam is another possibility for *Stenotrophomonas* infections offered in these guidelines, which was proposed before aztreonam/avibactam was widely accessible [8].

In this retrospective study, in vitro susceptibility of the most frequently detected non-fermenting Gram-negative bacilli (*Pseudomonas aeruginosa*, *Acinetobacter* sp., *Stenotrophomonas maltophilia*, and *Burkholderia cepacia* complex) against cefiderocol and aztreonam/avibactam was determined. For comparison with the in vitro effectiveness of commonly used antibiotics, the susceptibility to trimethoprim/sulfamethoxazole was also tested if applicable. There were two exceptions in susceptibility determination: *Acinetobacter* sp., for which aztreonam/avibactam was not tested, and *Pseudomonas aeruginosa*, for which trimethoprim/sulfamethoxazole was not tested, due to low or no expected antimicrobial activity. The isolates were obtained from clinical samples of patients hospitalized in General University Hospital in Prague, providing centralized care within a large hemato-oncology department.

## 2. Results

From 2022 to 2024, a total of 94 isolates of Gram-negative non-fermenting rods were collected. During this period, only 24 strains of *Stenotrophomonas maltophilia* and 10 strains of *Burkholderia cepacia* complex were detected in clinically significant samples. Furthermore, 30 isolates of *Pseudomonas aeruginosa* and 30 isolates of *Acinetobacter* sp. were included; 15 strains from both these groups were resistant to carbapenems, and 15 strains were susceptible.

**Figure 1:**
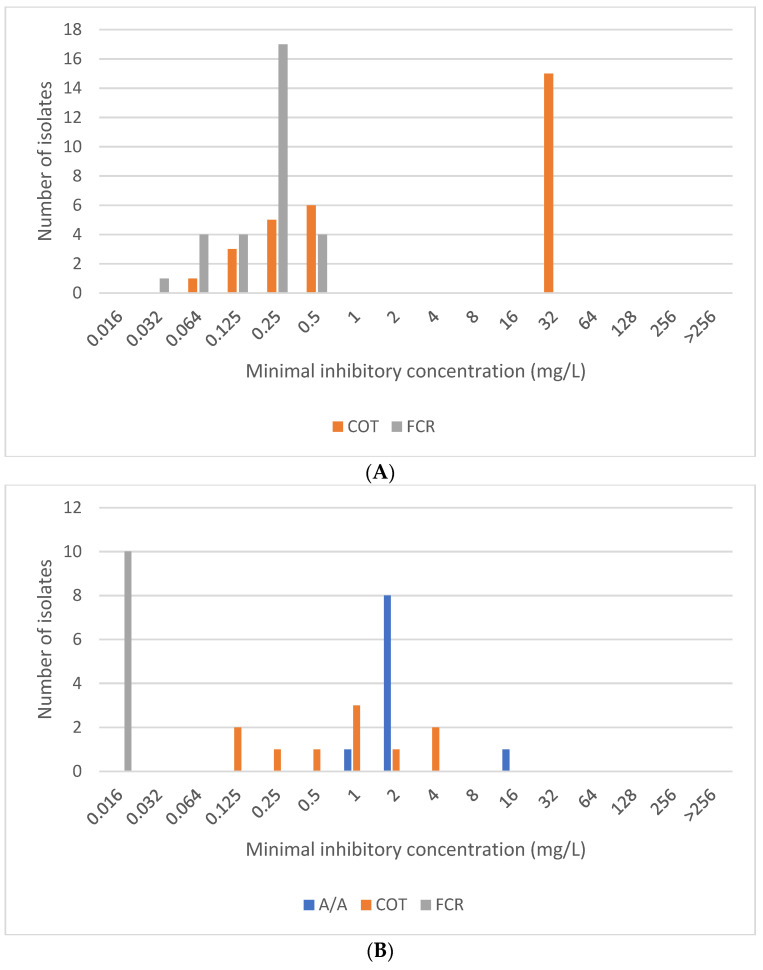

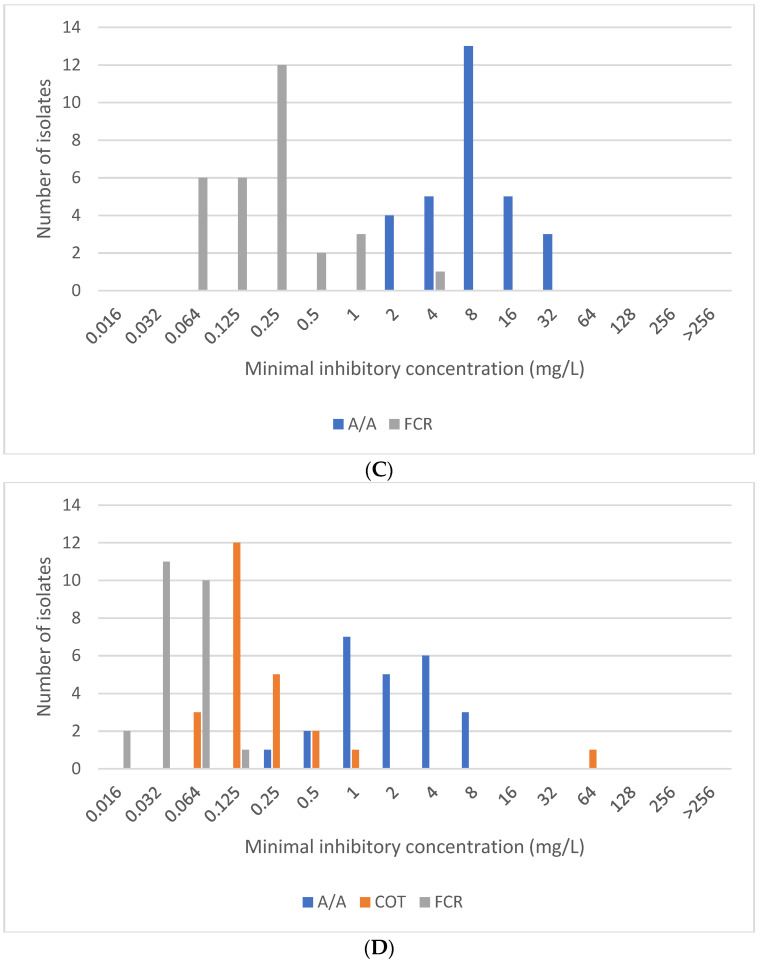
(**A**) Trimethoprim/sulfamethoxazole (COT) and cefiderocol (FCR) MIC distribution for *Acinetobacter* sp. Breakpoint for COT is 4 mg/L, and ECOFF for FCR is 0.5 mg/L. (**B**) Aztreonam/avibactam (A/A), trimethoprim/sulfamethoxazole (COT), and cefiderocol (FCR) MIC distribution for *Burkholderia cepacia* complex. Breakpoint for COT is 2 mg/L, ECOFF for A/A is 8 mg/L, and for FCR, 0.125 mg/L. Breakpoints and ECOFFs established for *S. maltophilia* were used. (**C**) Aztreonam/avibactam (A/A) and cefiderocol (FCR) MIC distribution for *Pseudomonas aeruginosa*. Breakpoint for FCR is 2 mg/L, and ECOFF for A/A is 16 mg/L. (**D**) Aztreonam/avibactam (A/A), trimethoprim/sulfamethoxazole (COT), and cefiderocol (FCR) MIC distribution for *Stenotrophomonas maltophilia.* Breakpoint for COT is 2 mg/L, ECOFF for A/A is 8 mg/L, and for FCR, 0.125 mg/L.

The minimum inhibitory concentration (MIC) distribution of all microorganism–antibiotic combinations and MIC_50_ and MIC_90_ calculated from total number of strains are shown in Figure 1A–D and Table 1, respectively.

### 2.1. Stenotrophomonas maltophilia

*S. maltophilia* isolates collected and tested in this study were generally susceptible to selected antimicrobial agents. One strain exhibited confirmed resistance to COT; however, most of the remaining strains (52.2%) demonstrated a quite low MIC value of 0.125 mg/L. MICs of FCR and A/A were all in the susceptible range, as defined by the ECOFFs, with FCR having a relatively narrow range of low values (0.016–0.125 mg/L), whereas A/A displayed a broader distribution with higher MIC values (0.25–8 mg/L).

### 2.2. Burkholderia cepacia Complex

According to EUCAST, no interpretative criteria are available for *B. cepacia* complex; therefore, for data evaluation, breakpoints and ECOFFs established for *S. maltophilia* were used. All tested isolates displayed very low MICs of FCR, supporting their classification as “susceptible”. An MIC higher than the adopted ECOFF for A/A (8 mg/L) was reported only in one strain, while the majority of the strains showed values several dilutions below this threshold. The widest range of MICs (0.125–4 mg/L) was observed for COT, with 2 isolates (20%) identified as resistant.

### 2.3. Acinetobacter sp.

In the tested group, resistance to carbapenems (CARB) directly related to resistance to COT (see Table 2). All *Acinetobacter* sp. isolates were susceptible to FCR (according to ECOFF), but 70% of them (n = 21) exhibited MICs on the ECOFF or just below. Susceptibility to A/A was not determined because there was no significant activity shown against *Acinetobacter* sp.

### 2.4. Pseudomonas aeruginosa

Among *P. aeruginosa* isolates, FCR MIC values ranged from 0.032 to 4 mg/L, with 40% of them inhibited at 0.25 mg/L. One isolate exceeded the breakpoint, indicating resistance. MIC values of A/A ranged from 1 to 32 mg/L, with a peak at 8 mg/L (43.3% isolates). Based on the 16 mg/L ECOFF, three isolates (10%) were classified as resistant to A/A, while the remaining isolates were within the susceptible range. One isolate demonstrated resistance to both FCR and A/A, but surprisingly, it was not proven to be a carbapenemase producer, even though it was resistant to carbapenems. On the other hand, confirmed carbapenemase producers were susceptible to both FCR and A/A (see Table 2). Notably, the majority of isolates exhibited A/A MICs just below the breakpoint.

## 3. Discussion

This retrospective monocentric study aimed to assess susceptibility of highly resistant non-fermenting Gram-negative bacilli (NFGNB)—incl. *Acinetobacter* sp., *Burkholderia cepacia* complex, *Pseudomonas aeruginosa*, and *Stenotrophomonas maltophilia*—to novel potentially active agents, such as cefiderocol (FCR) and aztreonam/avibactam (A/A). Susceptibility to trimethoprim/sulfamethoxazole (COT) was also determined because it remains the treatment of choice for most of these pathogens, either in monotherapy or combination [8].

Non-fermenting Gram-negative rods represent a significant clinical challenge, particularly in immunocompromised patients such as those undergoing hemato-oncological treatment and individuals with cystic fibrosis. NFGNB are also recognized as significant pathogens in ventilator-associated pneumonia and hospital-associated sepsis. These pathogens are often intrinsically resistant to multiple antibiotics and capable of acquiring additional resistance mechanisms. Moreover, in many clinical situations, it can be difficult to distinguish true infection from mere colonization, which complicates therapeutic decision-making. Their ability to persist in hospital environments and form biofilms further complicates eradication. The limited therapeutic options and variability in susceptibility profiles make the selection of effective antimicrobial therapy difficult, which may lead to higher morbidity and mortality. Continuous surveillance and adequate susceptibility testing are therefore essential for optimizing treatment strategies and improving patient outcomes [1,6,9].

The interpretation of antimicrobial susceptibility, especially in less common NFGNB, is challenging, mainly because the available guidelines are limited and sometimes inconsistent. The majority of criteria still in use are available for *Pseudomonas* sp., although many of these have been gradually phased out in recent years. In the case of *Acinetobacter* sp., several breakpoints remain (e.g., carbapenems and aminoglycosides). For *Stenotrophomonas maltophilia*, EUCAST provides a breakpoint only for COT; however, the original “susceptible” interpretation has been revised to “susceptible, increased exposure”. Additional agents can be interpreted according to CLSI guidelines, which are more commonly used in regions outside Europe. Lastly, due to insufficient supporting data, EUCAST has not published any breakpoints for *Burkholderia cepacia* complex. CLSI, however, offers breakpoints for several agents, including beta-lactams, minocycline, levofloxacin, and COT [10,11].

*Acinetobacter* sp. are opportunistic pathogens frequently linked to ventilator-associated pneumonia and bloodstream infections, particularly in critically ill patients. Their clinical management is complicated by extensive antimicrobial resistance, including resistance to carbapenems and limited options among traditional agents, underscoring the need for novel therapies and robust stewardship programs [12].

*Stenotrophomonas maltophilia* is an occasional cause of nosocomial infections, especially in patients with malignancies or prolonged hospitalization, and despite its intrinsic resistance to many antibiotic classes, it typically remains susceptible to COT, which remains the first-line treatment [13]. Several authors also suggest a role of *S. maltophilia* in lung adenocarcinoma progression [14,15].

*Burkholderia cepacia* complex poses a significant threat to individuals with cystic fibrosis and immunosuppressed hosts due to its ability to persist in hospital environments and its intrinsic resistance to multiple antimicrobials, limiting therapeutic choices and complicating eradication strategies [16].

*Pseudomonas aeruginosa* is a leading pathogen in healthcare-associated infections, particularly in patients with structural lung disease or indwelling devices; its capacity to develop multidrug resistance through both chromosomal and acquired mechanisms makes it a major challenge in clinical microbiology, often necessitating the use of novel beta-lactam/beta-lactamase inhibitor combinations or other last-line agents [8].

The role of novel antibiotics in therapy of infections caused by NFGNB has recently been widely discussed. Cefiderocol was originally considered a breakthrough antimicrobial agent against highly resistant Gram-negative bacteria. It promised to be effective even against the most problematic pathogens, such as metallo-beta-lactamase producers. This optimism was later tempered by clinical experience. The resistance to FCR quickly increased, especially in the most worrying organisms like NDM-producing Enterobacterales. A study assessing susceptibility of Gram-negative bacteria to FCR across Europe reported that 48.6% of NDM-producing Enterobacterales displayed resistance (probably due to NDM-1 production) [17].

However, FCR shows great potential for treatment of infections caused by NFGNB. A Taiwanese study assessed susceptibility patterns of multi-drug-resistant NFGNB against FCR and comparators, which showed excellent results for FRC: susceptibility of difficult-to-treat (DTR) *P. aeruginosa* and *B. cepacia* complex was 100%, and multi-drug-resistant *S. maltophilia* and *Acinetobacter* sp. displayed 97.9% and 94.4% susceptibility, respectively [18].

Combination of aztreonam and avibactam shows good stability and clinical cure rates for carbapenemase-producing Enterobacterales, although its effect on NFGNB is still to be fully assessed [19]. The already-mentioned Taiwanese study determined A/A susceptibility, with 86.7% of *B. cepacia* complex isolates susceptible. However, DTR *P. aeruginosa* exhibited only 7.1% susceptibility [18]. Furthermore, in a study from the USA, the authors examined 200 *S. maltophilia* isolates and reported 99.5% susceptibility to A/A [20]. The same team also reported on data from Europe, Asia, and Latin America: 99.5% of strains in total, as well as 100% of COT-resistant isolates, were susceptible to A/A [21].

Although COT is a relatively old antibiotic, it remains a cornerstone in the treatment of infections caused by NFGNB. Its efficacy is particularly notable against *Stenotrophomonas maltophilia* [21]. Barrasa et al. emphasize the importance of COT as a first-line agent in critically ill patients with *S. maltophilia* infections, while also highlighting the necessity of performing susceptibility testing due to regional differences in resistance patterns between the USA and Europe [13]. In the case of *Burkholderia cepacia* complex, COT also shows substantial activity, with susceptibility rates typically reported between 80% and 100%, averaging around 87–89% [16,22]. Conversely, *Acinetobacter baumannii* generally demonstrates poor susceptibility to COT [23].

However, COT has been associated with bone marrow suppression, potentially leading to pancytopenia, particularly with prolonged use or in vulnerable populations. This adverse effect is of special concern in haemato-oncology patients, who often have already compromised hematopoiesis. This further supports the rationale for including novel antibiotics such as FCR and A/A into treatment strategies in this high-risk patient group [24,25].

In this study, *S. maltophilia* demonstrated promising results. This pathogen is typically intrinsically resistant to many antimicrobial agents. Beta-lactam antibiotics, including carbapenems, are hydrolyzed by L1 metallo-beta-lactamase, which does not affect aztreonam. However, aztreonam is hydrolyzed by L2 serine beta-lactamase, which can be inhibited by avibactam. This suggests that the combination of aztreonam with avibactam may overcome the activity of both L1 and L2 and could be a promising treatment option [26]. Our findings support this, as A/A in vitro was effective against all tested *S. maltophilia* isolates (according to EUCAST ECOFF 8 mg/L). These results are consisted with several previous studies reporting that nearly 100% of *S. maltophilia* strains were inhibited at concentrations ≤8 mg/L [20,27]. Nonetheless, Calvopiña et al. suggest that avibactam may be slowly inhibited by L1, potentially reducing its effectiveness in strains with L1 hyperproduction [28]. The MIC values of cefiderocol were considerably low for *S. maltophilia* in this study, with an MIC_90_ value of 0.064 mg/L, which was supported by countless studies around the globe [29,30,31]. An Italian study reported 100% susceptibility to FCR in comparison with colistin, ceftazidime/avibactam, and ceftolozane/tazobactam, resulting in rates of 69%, 41%, and 37%, respectively [32]. There was only one strain of *S. maltophilia* demonstrating resistance to trimethoprim/sulfamethoxazole, which is also in correlation with previous studies, such as Sader et al., who reported 96.3% COT susceptibility (1287 isolates) [21].

The most effective agent against *Burkholderia cepacia* complex in this study was FCR, as the MIC of 100% isolates was 0.016 mg/L, which is several dilutions below ECOFF, adopted from EUCAST interpretative criteria for *S. maltophilia*. This suggests great efficacy of FCR towards this pathogen. Our findings are consistent with the susceptibility data from the SENTRY surveillance program involving isolates from across Europe and North America; DeJonge et al. collected data about 160 strains with MIC_50_ and MIC_90_ values of 0.06 mg/L and 0.5 mg/L, respectively [33]. Another study collected strains as a part of SIDERO-WT multinational surveillance program and compared the susceptibility data of total number of isolates (n = 425) with meropenem non-susceptible ones (n = 184) showing 94.8% and 92.9% susceptible to FCR, respectively. The same study also determined susceptibility of *B. cepacia* complex to A/A, although there was no difference between total (n = 101) and meropenem non-susceptible isolates (n = 27): MIC_50_ and MIC_90_ of 4 mg/L and 8 mg/L, respectively. If the identical interpretation criterion in this study (EUCAST ECOFF for *S. maltophilia*) were used, ≈90% of isolates would be susceptible to A/A. For comparison, our results displayed 90% of isolates (1/10) inhibited with MIC ≤8 mg/L, while there were no meropenem-resistant strains detected. Similarly, meropenem non-susceptibility did not affect the susceptibility of *B. cepacia* complex to COT; in the SIDERO-WT study, the values were comparable (approx. 80% susceptible) [34].

All carbapenem-resistant strains of *Acinetobacter* sp. (CRAB) identified in our study also exhibited resistance to COT, reflecting local epidemiological patterns. It was published that the carbapenem resistant isolates in this region generally belong to epidemic clone II (ECII), which is associated with resistance to multiple other antimicrobial agents [35]. Globally, *Acinetobacter* sp. resistance rates to COT vary widely across different regions, reflecting diverse antimicrobial usage patterns and other factors. A study by Chinese authors reports 73.4% of CRAB strains resistant to COT [36]; another study from Turkey shows COT resistance of 60% among CRAB [23]; finally, a Greek study compares resistance rates during the years and displays 61.7%, 98.2%, and 85.7% in 2021, 2022, and 2023, respectively [37]. For cefiderocol, all tested *Acinetobacter* sp. strains belonged to the susceptible category; however, most MIC values were very close to the ECOFF (0.5 mg/L), suggesting a possible shift toward reduced susceptibility and a potential risk for the development of resistance. In the SIDERO-WT study, FCR susceptibility of 255 *A. baumannii* strains was determined. The results indicated that, if the ECOFF value used in our study were applied, 14.1% (n = 36) of isolates would be categorized as resistant [38]. Furthermore, a study analyzing 501 *Acinetobacter* sp. isolates sourced across Europe presented 7.6% resistance. Surprisingly, only 10% of these FCR resistant isolates were also carbapenem resistant [39].

The most effective agent for *P. aeruginosa* was cefiderocol. There was only one (1/30) strain resistant in our cohort, although its MIC value was only one dilution above breakpoint. Santerre Henriksen et al. also report high efficacy of FCR against *P. aeruginosa* (98.9%, 10/950), incl. carbapenem-resistant strains (97.8%, 4/139). The authors also analyzed FCR resistant strains and concluded that all of the isolates had alterations in either *piuA* or *piuC* genes or both [39]. *PiuA* encodes a siderophore uptake receptor, and *piuC* encodes a hydroxylase enzyme contributing to the siderophore-mediated iron acquisition [40]. Furthermore, according to the SIDERO-WT study, which determined FCR susceptibility for 375 *P. aeruginosa* isolates, only three (0.8%) isolates had MIC > 2 mg/L and therefore were deemed resistant [38]. Aztreonam/avibactam also shows overall efficacy, but with relatively high MIC values: MIC_50_ of 8 mg/L, MIC_90_ 16 mg/L. Very similar results were reported by Santerre Henriksen, with MIC_90_ > 8 mg/L [39], and Cercenado et al., with 32 mg/L [38].

In our study, carbapenemase production did not affect the susceptibility of *P. aeruginosa* to aztreonam/avibactam, considering all carbapenemase producers were categorized as susceptible. In contrast, none of the strains resistant to A/A (n = 3, 10%), including one strain that was resistant to both A/A and FCR, were found to produce carbapenemases. Notably, one of these strains remained susceptible to carbapenems. According to Karlowsky et al., the distribution of A/A MICs in *P. aeruginosa* is similar when comparing the whole cohort of isolates (n = 11,842) and metallo-beta-lactamase producers (n = 452): MIC_90_ 32 mg/L for both [41]. Accordingly, the resistance of *P. aeruginosa* to A/A has not been attributed to metallo-beta-lactamases but rather to mutations in Pseudomonas-derived cephalosporinase (PDC), horizontally acquired non-OXA-48 OXA beta-lactamases, and reduced antibiotic permeability, including mutations in outer membrane porins and overexpression of efflux pumps [42,43].

This study has several limitations to consider. First, it was conducted in a single center, which may not fully reflect the epidemiological and microbiological variability present in other regions or healthcare settings. Second, its retrospective design carries the risk of selection and information bias. Furthermore, the number of included isolates was relatively small, particularly for *Burkholderia cepacia* complex, because this particular pathogen is not often isolated in the setting of the study. This fact restricts the robustness of conclusions drawn for this group of microorganisms. Despite these limitations, the study provides important insights into antimicrobial susceptibility patterns of non-fermenting Gram-negative bacilli against new prospective antimicrobial agents and highlights areas for future investigations.

## 4. Materials and Methods

A retrospective monocentric study was conducted on 94 isolates of non-fermenting Gram-negative bacilli (NFGNB), including 30 isolates of *Pseudomonas aeruginosa*, 30 isolates of *Acinetobacter* sp., 24 isolates of *Stenotrophomonas maltophilia*, and 10 isolates of *Burkholderia cepacia* complex. The strains were sourced in 2022–2024 from clinically valid samples (blood cultures, lower respiratory tract samples, abdominal fluids) of patients hospitalized in a university hospital with approx. 1500 beds. The study includes all *S. maltophilia* and *B. cepacia* complex isolates identified in clinically significant samples during the observed period, while 30 isolates each of *P. aeruginosa* and *Acinetobacter* sp.—15 carbapenem-resistant and 15 carbapenem-susceptible—were selected at random.

The susceptibility of selected NFGNB isolates was assessed by determining minimum inhibitory concentrations (MIC) of cefiderocol (FCR), aztreonam/avibactam (A/A), and trimethoprim/sulfamethoxazole (co-trimoxazole, COT). Categories “susceptible” and “resistant” were interpreted according to European Committee on Antimicrobial Susceptibility Testing (EUCAST) breakpoints, where available [10]. In addition, the efficacy of the tested agents was evaluated by analyzing the MIC distribution and calculating the MIC_50_ and MIC_90_ values, defined as the lowest concentrations of the antimicrobial agents required to inhibit 50% and 90% of isolates, respectively.

### 4.1. Susceptibility Testing

MICs were determined using the gradient strip method with MIC Test Strip (MTS, Liofilchem, Roseto degli Abruzzi, Italy) according to the instructions of the manufacturer. The bacterial suspensions were adjusted to a turbidity equivalent of 0.5 McFarland standard and inoculated on Mueller Hinton agar for Etest^®^ (MHE agar; bioMérieux SA, Marcy l’Etoile, France). MIC strips were then applied to the inoculated plates, which were subsequently incubated at 37 °C for 24 h [44].

#### 4.1.1. Quality Control

To ensure methodological accuracy, quality control was performed using the following strains according to EUCAST: *Escherichia coli* ATCC 25922 (FCR, A/A, COT), *Klebsiella pneumoniae* ATCC 700603 (A/A), and *Pseudomonas aeruginosa* ATCC 27853 (FCR). These control strains were tested in parallel with clinical isolates during each antimicrobial susceptibility testing procedure [45].

#### 4.1.2. Carbapenemase Production

Each carbapenem-resistant strain of *Acinetobacter* sp. and *B. cepacia* complex, as well as each strain of *P. aeruginosa* resistant to both carbapenems and ceftolozane/tazobactam, was tested for carbapenemase production. *S. maltophilia* isolates were not tested due to their resistance to carbapenems mediated by L2 beta-lactamase [21]. Carbapenemase production was assessed using the Carba-5 lateral flow immunoassay (NG Biotech, Guipry-Messac, France). All results, including negative findings, were subsequently confirmed by the National Reference Laboratory for Antibiotics at the National Institute of Public Health.

#### 4.1.3. Notes on the Result Interpretation

Interpretation criteria for NFGNB and the tested antibiotics are largely lacking. In this study, the results were interpreted according to EUCAST breakpoints where available. EUCAST currently provides breakpoints only for the following combinations: FCR in *P. aeruginosa*, COT in *Acinetobacter* sp., and COT in *S. maltophilia*. In the absence of species-specific breakpoints, epidemiological cutoff values (ECOFFs) were applied. Where neither breakpoints nor ECOFFs were available, interpretative criteria for closely related species were used. An overview of the specific criteria applied for each species–antibiotic combination is provided in Table 3 [10].

## 5. Conclusions

Novel antibiotics such as aztreonam/avibactam and cefiderocol offer encouraging new options for treating infections caused by inherently multidrug-resistant non-fermenting Gram-negative bacilli, including *Pseudomonas aeruginosa*, *Stenotrophomonas maltophilia*, *Acinetobacter baumannii*, and *Burkholderia cepacia* complex. In our in vitro testing, both agents showed promising activity against a broad spectrum of isolates, highlighting their potential to manage these particularly difficult-to-treat pathogens. These antibiotics may help fill critical gaps in current antimicrobial therapy, especially where standard treatments fail or are unavailable. However, resistance to even these newer agents has already begun to emerge. Continued surveillance is crucial to track resistance trends and guide the clinical use of these antibiotics. Further research is needed to confirm our findings and support evidence-based treatment strategies.

## Figures and Tables

**Table 1:** MIC distribution and percentage of antibiotic resistance.

		*Acinetobacter* sp. (*n* = 30)	*Burkholderia cepacia* Complex (*n* = 10)	*Pseudomonas* *aeruginosa* (*n* = 30)	*Stenotrophomonas maltophilia* (*n* = 24)
A/A	**MIC_50_ (mg/L)**	NT	2	8	2
	**MIC_90_ (mg/L)**	NT	2	16	8
	**range (mg/L)**	NT	1 to 16	2 to 32	0.25 to 8
	**% resistant (*n*)**	**NT**	**10 (1)**	**10 (3)**	**0 (0)**
COT	**MIC_50_ (mg/L)**	0.5	1	NT	0.125
	**MIC_90_ (mg/L)**	32	4	NT	0.5
	**range (mg/L)**	0.064 to 32	0.125 to 4	NT	0.064 to 64
	**% resistant (*n*)**	**50 (15)**	**20 (2)**	**NT**	**4.2 (1)**
FCR	**MIC_50_ (mg/L)**	0.25	0.016	0.25	0.032
	**MIC_90_ (mg/L)**	0.5	0.016	1	0.064
	**range (mg/L)**	0.032 to 0.5	0.016 to 0.016	0.064 to 4	0.016 to 0.125
	**% resistant (*n*)**	**0 (0)**	**0 (0)**	**3.3 (1)**	**0 (0)**

A/A aztreonam/avibactam; COT, trimethoprim/sulfamethoxazole (co-trimoxazole); FCR, cefiderocol; MIC, minimum inhibitory concentration; NT, not tested.

**Table 2:** Susceptibility rates of *Pseudomonas aeruginosa* and *Acinetobacter* sp. against aztreonam/avibactam, trimethoprim/sulfamethoxazole, and cefiderocol according to resistance phenotype.

	Antibiotic		
	Aztreonam/Avibactam	Trimethoprim/Sulfamethoxazole	Cefiderocol
***Pseudomonas aeruginosa*** **(*n* = 30)**			
Carbapenem—susceptible (*n* = 15)	93% (14/15)	NA	100% (15/15)
Carbapenem—resistant Carbapenemase—negative (*n* = 10)	80% (8/10)	NA	90% (9/10)
GES (*n* = 3)	100% (3/3)	NA	100% (3/3)
VIM (*n* = 1)	100% (1/1)	NA	100% (1/1)
NDM (*n* = 1)	100% (1/1)	NA	100% (1/1)
***Acinetobacter*** **sp. (*n* = 30)**			
Carbapenem—susceptible (*n* = 15)	NA	100% (15/15)	100% (15/15)
Carbapenem—resistant Carbapenemase—negative (*n* = 15)	NA	0% (0/15)	100% (15/15)

NA, not applicable.

**Table 3:** Interpretative criteria for MIC determination according to EUCAST 15.0.

		*Acinetobacter* sp.	*Pseudomonas* *aeruginosa*	*Stenotrophomonas maltophilia*	*Burkholderia cepacia* Complex
MIC breakpoints (mg/L)	A/A	NA	16	8	8
	COT	4	NA	2	2
	FCR	0.5	2	0.125	0.125

A/A aztreonam/avibactam; COT, trimethoprim/sulfamethoxazole; FCR, cefiderocol; NA, not applicable. Yellow fields illustrate epidemiological cut-off values provided by EUCAST in absence of breakpoints; green fields illustrate values according to interpretative criteria for Stenotrophomonas maltophilia.

## Data Availability

The original contributions presented in the study are included in the article. Further inquiries can be directed to the corresponding authors.
